# Application of human cardiac organoids in cardiovascular disease research

**DOI:** 10.3389/fcell.2025.1564889

**Published:** 2025-03-31

**Authors:** Hongyan Zhang, Peng Qu, Jun Liu, Panke Cheng, Qian Lei

**Affiliations:** ^1^ Department of Anesthesiology, Chengdu Wenjiang District People’s Hospital, Chengdu, China; ^2^ Department of Anesthesiology, Sichuan Provincial People’s Hospital, School of Medicine, University of Electronic Science and Technology of China, Chengdu, China; ^3^ Institute of Cardiovascular Diseases and Department of Cardiology, Sichuan Provincial People’s Hospital, School of Medicine, University of Electronic Science and Technology of China, Chengdu, China; ^4^ Ultrasound in Cardiac Electrophysiology and Biomechanics Key Laboratory of Sichuan Province, Chengdu, China

**Keywords:** human cardiac organoids, cardiovascular disease, clinical translation, disease models, drug screening

## Abstract

With the progression of cardiovascular disease (CVD) treatment technologies, conventional animal models face limitations in clinical translation due to interspecies variations. Recently, human cardiac organoids (hCOs) have emerged as an innovative platform for CVD research. This review provides a comprehensive overview of the definition, characteristics, classifications, application and development of hCOs. Furthermore, this review examines the application of hCOs in models of myocardial infarction, heart failure, arrhythmias, and congenital heart diseases, highlighting their significance in replicating disease mechanisms and pathophysiological processes. It also explores their potential utility in drug screening and the development of therapeutic strategies. Although challenges persist regarding technical complexity and the standardization of models, the integration of multi-omics and artificial intelligence (AI) technologies offers a promising avenue for the clinical translation of hCOs.

## 1 Introduction

### 1.1 Epidemiological and therapeutic challenges of cardiovascular diseases

Cardiovascular disease (CVD) encompasses a range of disorders affecting the circulatory system and constitutes a significant contributor to the global disease burden, with a reported prevalence of 523 million cases (Roth et al., 2020; Tsao et al., 2022). According to data published by the WHO, approximately 17 million individuals succumb to cardiovascular-related diseases each year, representing nearly 31% of global deaths. Projections indicate that by 2030, the annual mortality from CVD will rise to an estimated 23.3 million individuals (Chen et al., 2023). The prevalence and incidence of CVD vary markedly in different countries and regions, but the overall trend suggests that the burden of these diseases is increasing (Yeh et al., 2010; Dyrbuś et al., 2019; Mosterd and Hoes, 2007; Kusumoto et al., 2019; Heeringa et al., 2006). In recent years, the global prevalence of CVD has shown an expansion towards younger age groups (Andersson and Vasan, 2018), which is closely related to the increase in unhealthy lifestyle habits and environmental factors. Despite significant advances in CVD therapies, the emergence of novel agents such as proprotein convertase subtilisin/kexin type 9 (PCSK9) inhibitors (LDL-cholesterol-lowering) (O'Donoghue et al., 2019) and sodium-glucose cotransporter 2 (SGLT2) inhibitors (endothelial function-improving) (Vallon and Verma, 2021) has redefined cardioprotection. By targeting distinct pathological pathways such as atherosclerosis via LDL-C reduction and endothelial dysfunction via glucuretic/anti-inflammatory effects, these drugs synergistically mitigate atherosclerotic progression and cardiovascular mortality. Contemporary animal models utilized in CVD research are typically genetically or surgically altered to manifest a spectrum of symptoms pertinent to specific diseases. While these models are instrumental in advancing research, the intrinsic species differences between humans and animals, coupled with the disparities at the organ and cellular levels, pose significant challenges in extrapolating animal study results to human clinical trials, often resulting in a clinical translational bottleneck (Wang et al., 2017). In contrast, the cultivation of human cardiac organoids (hCOs) in laboratory settings offers a promising avenue to bridge the gap between animal studies and clinical research (Keung et al., 2019). This approach provides an innovative method for investigating the physiological functions and pathological mechanisms of human heart diseases. As a next-generation model, hCOs largely preserve the biological properties and functions of *in vivo* cells, thereby significantly advancing CVD research.

### 1.2 Development and application of hCOs technology in cardiac research

The origin of organoid technology can be traced back to cell culture technology in the 1990s (Folkman and Moscona, 1978), and early cardiac research relied on two-dimensional (2D) cell culture models, which, although playing an important role in basic research, often failed to realistically reproduce the complex physiological environment and function of the heart (Schaaf et al., 2011). In contrast, this field has developed significantly in recent years with advances in microfluidics and biomaterials science (Nahak et al., 2022).The application of organoid technology in cardiac research has covered a wide range of aspects, including the establishment of cardiac disease models, drug screening and toxicity assessment, cardiac development and regeneration studies. For example, by combining oxygen diffusion gradients and norepinephrine stimulation, hCOs can accurately mimic myocardial infarction and hypoxia-enhanced doxorubicin cardiotoxicity in humans, which provides a valuable tool for drug screening and development (Richards et al., 2020). In addition, Plansky Hoang et al. identified a new combination of biomaterial-based cell patterning and stem cell organoid engineering technology to create 3D cardiac microchambers from 2D induced pluripotent stem cells (iPSCs), which can mimic early cardiac development and be used as an embryotoxicity screening assay (Hoang et al., 2018). Lee et al. (2020) developed a hCOs model derived from mouse embryonic stem cells to study the spatiotemporal regulation mechanisms of the heart, which was able to self-organize to form tissue structures similar to the real heart under specific conditions, with cardiac tissue structure of a real heart with myocardial contraction and action potential properties, providing a promising research tool for cardiac development and drug testing. In addition, organoid technology has been widely used for screening and toxicity testing of new drugs, providing more accurate drug response data than traditional cell culture models. Kenji Kosuke et al. cultured miniature colonic organoids in 96-well plates and successfully screened compounds for 2000 potential drugs (Kozuka et al., 2017). In conclusion, organoid technology has gained increasing importance in biomedical research as an important tool for exploring organ function, disease mechanisms and drug screening and development (Hofbauer et al., 2021; Ma et al., 2021).

## 2 Overview of hCOs

### 2.1 Definition and characterization

Organoids are three dimensional (3D) cellular aggregates generated *in vitro* from stem cells or human induced pluripotent stem cells (hiPSCs). hCOs, which currently lack a standardized definition, are typically 3D microstructures that spontaneously arise from hiPSCs or other cardiac-related cells, partially mimicking the structure or function of cardiac tissues. These organoids encompass major cardiac cell types. These structures mimic the development, architecture, and function of the heart *in vitro* (Kim et al., 2020; Voges et al., 2017). Cardiac organs have many features: (1) 3D structure: Compared with traditional 2D cell culture models, 3D models can better reproduce the natural structure of cardiac tissues, including the arrangement of myocardial layers and the formation of vascular networks (Zhang and Radisic, 2017; Thavandiran et al., 2013), This is primarily due to the ability of 3D models to provide mechanical and biochemical cues that regulate cardiomyocyte alignment, extracellular matrix (ECM) deposition, and vascular development (Beauchamp et al., 2015). Furthermore, 3D hCOs create physiologically relevant cell-cell and cell-matrix interactions, supporting the maturation of cardiomyocytes and the development of functional cardiac tissue. These features enable hCOs to better mimic the dynamic microenvironment of the heart; (2) Cellular heterogeneity: A notable feature of hCOs is their high cellular heterogeneity, which allows them to mimic the complex cellular diversity of native cardiac tissue. hCOs typically contain various cell types, including cardiomyocytes, endothelial cells, fibroblasts, and cardiac progenitor cells (Ma et al., 2021; Ronaldson-Bouchard et al., 2018). This multicellular composition not only supports the structural integrity of cardiac tissue but also promotes the formation of a functional microenvironment (Ergir et al., 2022); (3) Functional analysis: weave-engineered cardiac patches promoted the functional maturation of human embryonic stem cell (hESC) derived cardiomyocytes (Zhang et al., 2013), utilized electrophysiological tests and other techniques to assess the functional properties of hCOs, including the excitability and conductivity of cardiomyocytes; (4) Disease modelling function: cardiac class organs can be used to simulate a variety of cardiac disorders. For example, Richards et al. (2020) demonstrated that hCOs can replicate the structural features of the heart after myocardial infarction, including the infarcted, marginal, and distal zones, making them a promising model for studying heart repair. Tiburcy et al. (2017) established a systematic approach for generating hCOs that closely mimic the structural and functional properties of the postnatal myocardium. Together, these studies highlight the potential of hCOs to serve as accurate models for both myocardial infarction and postnatal heart function, providing valuable insights for therapeutic development.

### 2.2 Different types of human cardiac organ models

HCOs can be categorized into several types, each with unique methods and applications:

• Embryoid Bodies (EBs)-derived Organoids: Burridge and Paul W. utilized embryoid bodies’ cardiac differentiation potential to generate 3D cardiac tissues under specific culture conditions, which has provided valuable insights into early cardiac development and cell differentiation (Burridge et al., 2014). These models offer a foundational approach to studying cardiogenesis.
•Cardiac Microtissues: Through bioprinting technology, the precise arrangement of cardiac cells and biomaterials has been used to create tissues with complex structures and functions. Lind et al. (2017) advanced this field by developing instrumented cardiac microphysiological devices, allowing for high-throughput drug screening, toxicity assessments, and investigations into cardiac contraction and development. This approach enhances the fidelity and versatility of cardiac models for therapeutic testing.
•Engineered Cardiac Tissue Blocks: These models involve the 3D culturing of cardiomyocytes or stem cells on biological scaffolds to create functional cardiac tissues. Tao et al. (2018) demonstrated the creation of cardiac structures by seeding adult rat cardiomyocytes onto fibrin gel matrices, providing a useful tool for disease modeling and drug screening.

Together, these diverse types of hCOs contribute to a more comprehensive understanding of cardiac function and disease, with specific applications in drug discovery, disease modeling, and regenerative medicine.

## 3 Methods for the preparation of human cardiac organs

**FIGURE 1:**
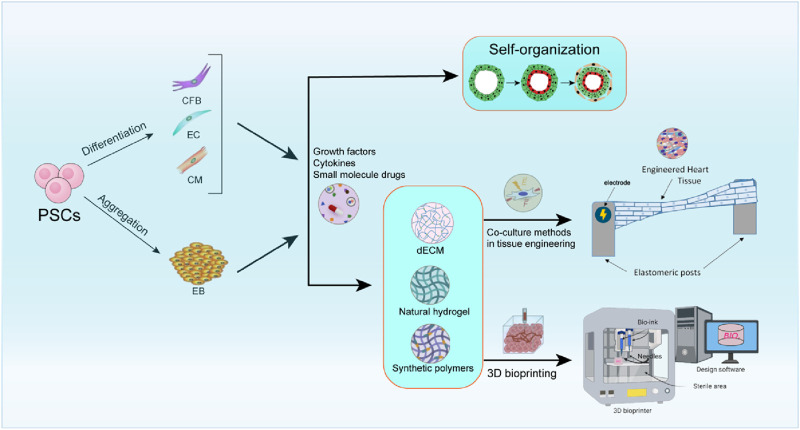
Overview of methods for the preparation of cardiac organoids. This includes scaffold-free self-organization methods, where cells assemble themselves to form cardiac tissue (Filippo Buono et al., 2020); tissue engineering co-culture methods, which combine different cell types to simulate the cardiac microenvironment; and 3D printing methods, which utilize precise bioprinting techniques to construct complex cardiac structures. Each of these approaches has unique advantages and contributes to advancements in cardiac regenerative medicine and disease modeling.

Currently, organoids are classified as scaffold-free self-organization structures and co-cultures incorporating active materials (as shown in Figure 1).

### 3.1 Self-organization in cardiac organoid generation

Self-organizing methodologies utilize stem cells with high differentiation potential alongside specific developmental signaling molecules to generate hCOs. These approaches elucidate cardiac development by directing stem cells toward the cardiac mesodermal lineage, promoting self-organization that generates diverse cell populations and enhances physiologically relevant cellular interactions critical for cardiac morphology and function (Yang et al., 2023). For example, Keung et al. (2019) was able to more realistically mimic the physiological and pharmacological properties of the human heart by using human hiPSCs to self-organize to generate organoid and tissue strips with ventricular features, providing a reliable model for drug screening and heart disease research, and this technological platform is particularly suited to the evaluation of drugs that affect myocardial contractility (i.e., positive and negative inotropic drugs). Recently, Hofbauer et al. (2021) developed a temporally regulated differentiation protocol utilizing key cardiogenic signaling pathway, encompassing both hESCs and hiPSCs. This method was facilitated through the incorporation of laminin 521/511, hPSCs demonstrated a self-assembly efficiency exceeding 90% into mesoderm, cardiac mesoderm, and beating cardiac progenitors within 2D cultures. Furthermore, these cells rapidly self-organized into beating luminal structures, termed “cardiac shapes,” in 3D non-adherent high-throughput cultures without the addition of exogenous extracellular matrix, also achieving 90% efficiency. These structures exhibited tissue characteristics reminiscent of myocardial nodules and intercalated discs. Electrophysiological assessments and calcium imaging were employed to evaluate the myocardial contractile function and electrical activity of the organoids. Lewis-Israeli et al. (2021) have successfully developed a time-regulated differentiation methodology that leverages key cardiogenic signaling pathways to facilitate the self-assembly of physiologically functional 3D hCOs. These organoids express a range of myocardial markers. This hCO model not only exemplifies the self-organization principles inherent in cardiac development but also offers a novel platform for the investigation of congenital heart disease and the screening of pharmaceutical compounds. Recently, Branco et al. (2022) successfully developed a self-organized multispectral embryonic organoid exhibiting early embryonic structures, such as primitive endoderm (PE), septal mesenchymal stromal cells, and liver buds, through the modulation of the WNT signaling pathway in induced hiPSCs. Richards et al. (2017) utilized hiPSCs in conjunction with cardiac fibroblasts to engineer biohybrid human heart-like structures. Firstly, scaffold-free self-organization techniques are constrained by inadequate nutrient and oxygen delivery, rendering them unsuitable for extensive clinical application. Secondly, these methods lack uniformity and do not permit precise regulation of cell proportions. Thirdly, self-assembled tissues exhibit greater fragility compared to those produced through conventional tissue engineering techniques, primarily due to a lack of adequate support materials and 3D structural integrity. Furthermore, self-assembled hCOs typically display immaturity. Therefore, additional research is needed to promote tissue and cell maturation in self-assembled hCOs.

### 3.2 Co-culture strategies in cardiac organoid development

Tissue engineering co-culture methodologies comprise three fundamental components: seed cells, scaffolds that provide structural support, and bioactive factors that modulate cellular behavior, all of which are integral to the development of 3D tissue and organ models (Borovjagin et al., 2017). The co-culture system significantly enhances the physiological relevance of heart organoids by simulating *in vivo* multicellular interactions. hiPSCs, with their robust proliferative capacity and patient-specific origin, have become the ideal seed cells in co-culture systems (Clevers, 2016). iPSC-derived hCOs can recapitulate the complete genomic features of the donor, providing an important tool for personalized drug testing and disease modeling (Yang et al., 2023).

Co-culture methodologies capitalize on the synergistic interactions between cardiomyocytes (CMs), endothelial cells (ECs), fibroblasts, and other cardiac cell types to recapitulate the complex cellular architecture of the heart.

In 2017, Polonchuk et al. (2017) have developed 3D *in vitro* models of the human heart, known as “cardiac spheroids,” by co-culturing human primary or iPSC-derived cardiomyocytes, endothelial cells, and fibroblasts in ratios that approximate those found *in vivo*. This approach successfully replicates the human cardiac microenvironment. Similarly, Mills et al. (2019) developed a 96-well high-throughput screening plate, the Heart-Dyno, for high-throughput screening of human pluripotent stem cell-derived heart organoids and optimization of their maturation conditions. This device promotes the formation of compact myofibrils and is capable of automatically analyzing the contraction force of heart organoids, significantly reducing the use of cells and reagents. The resulting hCOs exhibit structural similarities to native cardiac tissue, including highly organized cardiomyocytes, stromal cells, endothelial microvascular networks, and epicardial layers. Under culture conditions that simulate the postnatal metabolic environment, the maturity of hCOs is further enhanced, providing an efficient tool for drug screening and disease modeling. More recently, Drakhlis et al. (2021) successfully generated complex, highly structured 3D heart-forming organoids (HFOs) by embedding hPSCs aggregates in Matrigel and directing cardiac differentiation through biphasic WNT pathway modulation using small molecules. HFOs consist of a myocardial layer lined with endocardial-like cells and surrounded by septum-transversum-like tissue. Additionally, they contain spatially and molecularly distinct anterior and posterior foregut endoderm tissues, along with a developing vascular network. The architecture of HFOs closely resembles aspects of early native heart anlagen, particularly before heart tube formation. This model provides a new perspective for studying human heart development mechanisms and congenital heart diseases.

Biological active factors regulate cell signaling pathways in co-culture systems, driving the self-organization and functional maturation of hCOs (Unal and West, 2020). As Hofbauer et al. (2021) demonstrated, WNT and ACTIVIN dosages during the early stages of mesoderm differentiation influence the self-organization of cardiac-like organs. This regulation affects the patterning and morphogenesis of CMs and EC profiles. Additionally, the specification and patterning of the EC layer within the cardiac mesoderm are directed by VEGF levels and Other growth factors (Iyer et al., 2012; Montessuit et al., 2006; Yang et al., 2014; Parikh et al., 2017; Lee et al., 2017).

In co-culture systems, scaffold materials for cardiac organoids serve a pivotal role, primarily by mimicking the natural extracellular matrix (ECM) to offer structural support, biochemical signals, and mechanical cues (Capulli et al., 2016) (Xia and Chen, 2022). Scaffold materials can be classified into two primary categories based on their origin and properties: natural biomaterials (e.g., hydrogels and decellularized ECM) and synthetic polymers (Sharma et al., 2019; Tian et al., 2015; Cho et al., 2021; Pok et al., 2013). 1)Natural biomaterials, including natural hydrogels and decellularized extracellular matrix (dECM), offer significant advantages such as excellent biocompatibility and the ability to mimic natural ECM components. Hydrogels, which are 3D network structures constructed from natural or synthetic polymers, are particularly notable for their tunable mechanical properties and degradation rates (Radhakrishnan et al., 2014). However, their relatively low mechanical strength limits their application in high-load environments such as cardiac and other mechanically demanding organoids (Li et al., 2024). Decellularized extracellular matrix (dECM) retains the structural and biochemical components of the natural ECM through decellularization techniques, making it a popular scaffold material in cardiac bioengineering (Yang et al., 2023; Zhang et al., 2022; Jang et al., 2017). Jang et al. (2017) used dECM derived from cardiac tissues containing stem cells to enable improved intercellular interactions and differentiation capacity of stem cells, to provide a cardiac-like microenvironment for pre-vascularized constructs, and to have a beneficial impact on cardiac repair. 2) synthetic polymers are frequently employed in the construction of engineered cardiac tissues (Unal and West, 2020; Coenen et al., 2018; Saludas et al., 2017; Reis et al., 2016). Due to their customizability and reproducibility, they play a pivotal role in cardiac organoid scaffolds. Pok et al. (2013) engineered a polycaprolactone scaffold integrated with a gelatin-chitosan hydrogel by amalgamating diverse scaffolding materials. This innovation facilitated the seeding and migration of cardiomyocytes within a biomimetic environment while ensuring adequate tensile strength. Gelmi et al. (2016) developed an electroactive composite scaffold composed of PLGA fibers and a polypyrrole coating. This material integrates electrical conductivity, mechanical driving capability, and a biomimetic topological structure, providing a dynamic physico-chemical environment for hiPSCs. The scaffold can provide both electrical and mechanical stimulation without exhibiting cytotoxicity, significantly enhancing the expression of cardiac biomarkers.

Although co-culture systems have made significant progress in cardiac organoid development, they still face a series of challenges that limit their widespread application. The main issues include insufficient cell maturation, vascularization defects, and the difficulty of standardizing co-culture systems. Currently, some solutions have been proposed. For example, external stimuli such as mechanical stretching (Chiou et al., 2016; Lu et al., 2021; Li et al., 2020) and electrical pulses (Heidi Au et al., 2009) have been shown to promote the maturation of cardiomyocytes. In addition, the introduction of pro-angiogenic factors, such as VEGF, effectively promotes the proliferation of EC, thereby forming functional vascular networks within the cardiac organoids (Li et al., 2024).

### 3.3 3D printing

The process of 3D bioprinting involves layer-by-layer positioning of biological agents within 3D layers of tissue, thereby accurately replicating complex geometries of the heart such as cardiac spheroids (Beauchamp et al., 2020; Giacomelli et al., 2020; Filippo Buono et al., 2020), cardiac patches (Zhang et al., 2013; Jackman et al., 2018; Gao et al., 2018), cardiac specimens (Feric et al., 2019), and cardiac rings (Goldfracht et al., 2020) for a various applications. 3D bioprinting technology has the capability to fabricate perfused vascular networks and diverse cardiac tissue structures (Borovjagin et al., 2017). Basara et al. (2021) prepared a bioink was formulated by combining human induced pluripotent stem cell-derived cardiomyocyte (hiPSC-CMs) with hCFs. This bioink was utilized to print the infarct border region, effectively replicating the infarcted area surrounded by healthy tissue, but it was not able to achieve physiologically accurate stiffness of myocardium. Lee et al. (2019) employed collagen as a bioink to fabricate the outer shell of a human ventricular model. This model demonstrated synchronized contraction and directional propagation of action potentials, with the ventricular wall exhibiting a 14% increase in thickness during contraction after 7 days. Additionally, the researchers produced three 28 mm collagen heart valves and improved their mechanical properties using a decellularized porcine heart valve fixation protocol. At the adult scale, the collagen valves had well-separated leaflets, were well-suited to handling in air, and demonstrated mechanical integrity and functionality. Gaetani and colleagues (Gaetani et al., 2012) demonstrated that microstructural tissue printing using a combination of alginate scaffolds with human fetal cardiomyocyte progenitors (hCMPCs) could be used to fabricate cardiac-derived patches with defined pore sizes and improved viability. A novel cardiogenic scaffold consisting of hCMPCs and hyaluronic acid/gelatin (HA/gel)-based biomaterials was created to advance the attachment and survival of the hCMPCs without compromising their growth and differentiation potential. Kawai et al. (2021) utilized hiPSCs to develop engineered heart tissue (EHT), which holds promise as a potential cardiac tissue replacement for heart failure treatment. To fabricate pulsatile catheters for patients with congenital heart disease, they employed hiPSC-COs and bio-3D printing to construct stentless tubular EHT (T-EHT) and transplante into the abdominal aorta and around the inferior vena cava (IVC) of NOG mice. Post-transplantation, the T-EHT was enveloped by the omentum, and the abdominal cavity was sutured closed upon completion of the procedure. Furthermore, to evaluate the functional performance of hiPSC-COs in comparison to T-EHTs, the hiPSC-COs were transplanted in proximity to the aorta and IVC, as well as injected into the subcutaneous tissues. The beating of T-EHTs in mice was observed 1 month after the transplantation procedure. In histological analyses, T-EHTs showed distinct myocardial striations and angiogenesis compared with hiPSC-COs. T-EHTs bio-3D printed showed better maturation *in vivo* than hiPSC-COs. These beating T-EHTs may serve as a conduit for patients with congenital heart disease, making T-EHT transplantation a therapeutic option. By integrating hiPSC-CMs, ECs, and ECM, Noor et al. (2019) created cardiac parenchymal tissue, blood vessels, and functional cardiac patches. Kupfer et al. (2020) fabricate vascular inlet and outflow constructs using optimized ECM protein bio-inks. Nonetheless, several challenges persist in this field.

Although promising, the field of 3D bioprinting for cardiac applications continues to face significant challenges, including achieving precise tissue mechanics, scalability, and long-term tissue functionality post-transplantation (Basara et al., 2021; Hwang et al., 2024; Mehrotra et al., 2020). Further advancements are needed to enhance the viability, maturity, and integration of printed tissues in clinical settings.

## 4 Application of hCOs in different cardiovascular disease models

**FIGURE 2:**
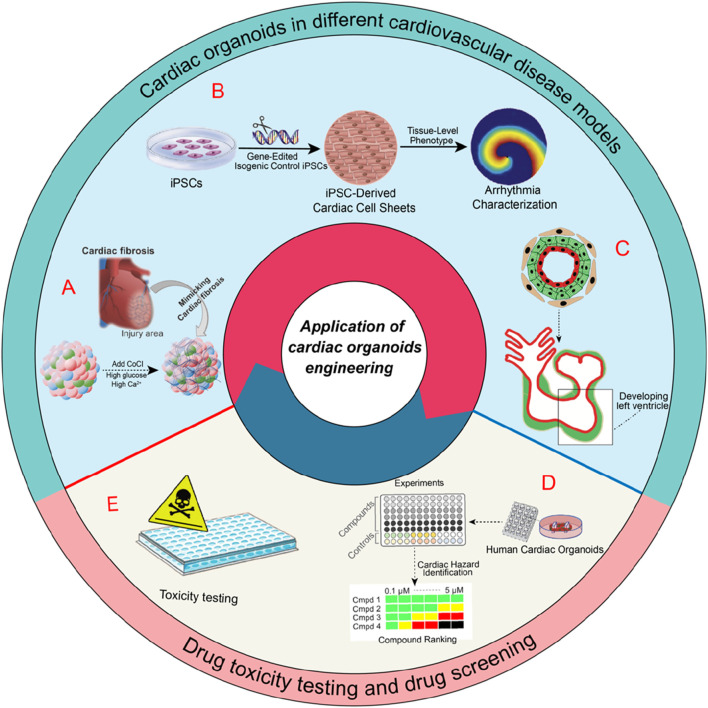
Applications of cardiac organoids in various cardiovascular disease models and their roles in drug toxicity testing and drug screening. A) schematic of an experiment mimicking heart disease in organoids via the IR injury mechanism occurring in the human adult heart, followed by the study of fibrogenesis (Song et al., 2024). B) cardiac organoids in arrhythmia research (Shinnawi et al., 2019). C) modeling heart chamber development and disease (Hofbauer et al., 2021). D) Illustrates the role of hCOs in drug screening for cardiovascular diseases. E) their application in drug toxicity testing.

Significant advancements have been achieved thus far in the development of platforms for cardiovascular modeling. hCOs provide extensive advantages for investigating cardiac function under pathological condition. In addition to myocardial ischemic injury, contractile dysfunction, and aberrant electrophysiological activity, these advancements may stimulate research in various fields of cardiac physiology (as shown in Figure 2).

### 4.1 Myocardial infarction

Organoid models of myocardial infarction or injury represent some of the earliest established hCO models for studying cardiovascular disease. Voges and his colleagues (Voges et al., 2017) developed hCOs by employing collagen-gel cultures of hPSC-CMs within circular molds. They successfully simulated acute myocardial infarction by subjecting the hCOs to cryoinjury, resulting in localized tissue damage. Richards et al. (2020) employed non-adhesive agarose hydrogel molds to construct a composite of hiPSC-CMs and non-cardiomyocytes, which were utilized to generate hCOs. Using transcriptomic, structural, and functional characteristics of myocardial infarction, the researchers successfully developed a non-genetic *in vitro* model of hCOs. This approach facilitated the replication of infarcts, border zones, and remote regions of the post-infarction heart.

Nevertheless, the aforementioned hCOs lack the ability to incorporate the full spectrum of heart cell types found *in vivo*. After myocardial infarction, immune and inflammatory cells are crucial to the initial response and the subsequent fibrotic response (Forte et al., 2018). Song et al. (2024) employed hiPSCs to generate self-organizing heart-like organoids (HOs). These organoids comprised cardiomyocytes, fibroblasts, and endothelial cells, effectively replicating the cellular composition of the human heart and the multicellular architecture of HCOs. The organoids were then exposed to hypoxia-induced ischemia and ischemia-reperfusion (IR) injury under controlled conditions, which resulted in a phenotype analogous to acute myocardial infarction. Furthermore, IR-exposed HOs showed significant cardiac fibrosis, collagen deposition, and electrophysiological abnormalities similar to heart disease. The results are significant for the advancement of 3D cardiac models and myocardial infarction models *in vivo*.

### 4.2 Heart failure

The key pathophysiological features of Heart failure (HF)include systolic and diastolic dysfunction, cardiomyocyte hypertrophy (Du et al., 2024), apoptosis, and neurohumoral activation (e.g., catecholamine overstimulation) (Ren et al., 2024). Despite advancements in therapeutic strategies, HF remains a leading cause of morbidity and mortality worldwide (Najjar et al., 2024), underscoring the urgent need for innovative preclinical models to study its mechanisms and develop targeted therapies. Similarly, Tiburcy et al. (2017) presented a method for developing hCOs derived from postnatal myocardium utilizing hiPSC-CMs and fibroblasts. To simulate human heart failure, they implemented a neurohumoral overstimulation protocol. hCOs models showed responses to chronic catecholamine toxicity, including systolic dysfunction, a diminution in positive force-frequency response, cardiomyocyte hypertrophy, cardiomyocyte apoptosis, and desensitization to adrenergic signaling, and the release of cardiac biomarkers. These responses collectively represent hallmark characteristics of heart failure. Significantly, the inhibition of β1-adrenergic and α1-adrenergic receptors by metoprolol and phenoxybenzamine partially or entirely mitigates the pathological phenotype associated with heart failure. However, immune cells are crucial in the pathophysiology of myocardial damage (Swirski and Nahrendorf, 2018). Nonetheless, using cardiac-like organoids are deficient in immune cells and immature cardiomyocytes. This deficiency presents a significant challenge in accurately replicating the functional roles of immune cells and mature CMs in these conditions within hCOs.

### 4.3 Arrhythmias

hCOs surpass 2D models in arrhythmia research by leveraging their enlarged surface area and 3D geometry to model reentrant circuits through spatially heterogeneous electrical propagation (Drakhlis et al., 2021). Therefore, hCOs are an excellent disease model for studying arrhythmias. Shinnawi et al. (2019) advanced the application of hCOs in conjunction with distinctive hiPSC-CM sheets. A patient-specific hiPSC-CM approach combines CRISPR/Cas9 genome editing technologies with organoids to replicate arrhythmic events. The study demonstrated that the increased susceptibility of reentrant arrhythmias in thin slices of cardiomyocytes derived from short QT syndrome (SQTS) hiPSCs was consistent with the heightened inducibility of ventricular fibrillation. The authors employed the hCOs model to analyze the role of novel antiarrhythmic drug candidates. The findings indicated that quinidine and dipyridamole resulted in the prolongation of action potentials and action potential duration, as well as the suppression of arrhythmias. This study offers evidence that hCOs can effectively generalize the phenotype of SQTS. Gold and colleagues (Goldfracht et al., 2020) engineered hCOs comprising chamber-specific hPSC-CMs, and the study suggests that atrial hCOs are promising models for studying recurrent arrhythmias and shows that flecainide and vernakalant can effectively terminate reentrant activity in these models.

Laksman et al. (2017) generated atrial-specific tissue models for pharmacological testing using hESC. Compared with ventricular-like cardiomyocytes, they generated atrial-like cardiomyocytes that expressed atrial-specific genes and had shorter action potential (AP) durations. Using optical labeling techniques, they generated atrial-like CM slices and observed fast-folding rotor patterns in these slices. Using hESC-derived atrial CM preparations, they then demonstrated that flecainide and dovetailed modulate the foldback arrhythmogenic rotor activation pattern, which explains their efficacy in treating and preventing atrial fibrillation. It is noteworthy, however, that these hCOs lack relative chamber-specific structures and the expression of ion channel-related genes differs from that of adults. hCOs represent a significant advancement in the modeling of arrhythmias, offering a platform to study disease-specific mechanisms, test novel therapeutics, and explore the electrophysiological properties of human cardiac tissues. However, to fully realize their potential, further efforts are needed to enhance their maturity, ion channel expression, and structural complexity, ensuring that these models can accurately replicate the full range of arrhythmic events observed in humans.

### 4.4 Hereditary and congenital heart disease

For several decades, the genetic underpinnings of familial cardiomyopathies, including hypertrophic and dilated cardiomyopathy, have been recognized, and congenital heart defects are identified as the most prevalent human birth anomalies. Nonetheless, the comprehension of the pathogenesis of these conditions is constrained by the current limitations in accurately replicating the human heart *in vitro*. hiPSC-derived cellular models exhibit numerous characteristics emblematic of human diseases, such as disease-specific gene expression profiles, altered cellular morphology, dysfunctional ion channel activity, and abnormal contractile behavior (Pré et al., 2022), making them valuable tools for investigating the genetic and congenital underpinnings of these conditions. Researchers are increasingly using multicellular hCOs to examine cell-cell interactions in disease pathogenesis, advancing disease research. Marini et al. (2022) used hiPSCs from patients with Duchenne Muscular Dystrophy (DMD) to self-organize into hCOs (DMD-hCOs) in long-term cultures, but the DMD-hCOs were not proliferative at the beginning. A novel mutation in myosin heavy chain 7 (E848G) in familial cardiomyopathies was investigated by Yang et al. (2018), in which hCOs were interconnected to a force transducer and a length controller to assess tissue tectonic forces. Importantly, hCOs derived from familial cardiomyopathy exhibited significantly less alignment compared to the broader hCO cohort. Additionally, hCOs carrying the E848G mutation demonstrated a marked reduction in systolic function, while diastolic function remained largely unaffected. These results hold greater physiological relevance as they successfully replicate the contraction of aligned myogenic fibers within a 3D system. Lewis-Israeli et al. (2021) reported a method for generating developmentally relevant human cardiac-like organs by self-assembly using hiPSCs. At the transcriptomic, structural, and cellular levels, cardiac-like organs are comparable to age-matched fetal heart tissue. They can effectively model features of congenital heart disease induced by pregestational diabetes, offering valuable insights into the molecular pathology of these conditions.

Hereditary and congenital heart diseases remain challenging to model accurately due to limitations in hCO maturation and structural complexity. Future research should focus on improving maturation, vascularization, and chamber-specific models to better replicate conditions like familial cardiomyopathies and congenital defects such as septal defects (Fang et al., 2023; Ching et al., 2022).

### 4.5 Cardiac development

hCOs can also be used to study the developmental biology of the embryo. Rossi and his colleagues (Rossi et al., 2021) successfully modeled the early stages of cardiac development, including the formation of a vascular-like network, the production of FHF/SHF, and the emergence of crescentic structures using axial-mode mouse gastrula-like proteins. A study by Wu et al. (2022) explored the effects of cadmium (Cd) on development of the heart in 2D and 3D using an *in vitro* embryonic model as well as cardiomyocytes in 2D and 3D, alongside a hCOs formation model that simulates early cardiac development. Their study conclusively demonstrated that exposure to Cd at a concentration of 0.6 mM resulted in a transient upregulation of the mesoderm-related transcription factors MESP1 and EOMES during the initial phase of mesoderm induction, followed by a downregulation during the later stages of heart induction. This effect was attributed to the inhibition of the Wnt/β-catenin signaling pathway. Branco et al. (2022) employed hiPSCs cultured in a 3D environment to promote the differentiation of proepicardial (PE) and epicardial cells through the modulation of WNT, BMP, and RA signaling pathways, thereby facilitating the formation of PE/STM/PFH-like organoids. These organoids were subsequently co-cultured with cardiomyocyte aggregates to produce cardiac-like organoids, termed engineered myocardial organoids. The findings from this study underscore the pivotal role of WNT and RA signaling in the canonical development of organoids, while also highlighting the active involvement of BMP4 signaling in the canonical development of liver bud-like cells. Mills et al. (2017) demonstrated that a shift in fatty acid metabolism plays a pivotal role in cardiac maturation by suppressing key proliferative pathways, particularly those involving β-catenin and YAP1. Their findings revealed that the proliferation impairment induced by fatty acid metabolism could be rescued through overexpression of β-catenin and YAP1 or pharmacological activation of these pathways using a small molecule (Compound 6.28). These results provide critical insights into the metabolic mechanisms underlying fatty acid metabolism-induced cell cycle arrest in cardiomyocytes, highlighting potential therapeutic strategies to modulate cardiac proliferation and maturation. Lewis-Israeli et al. (2021) investigated the impact of preconception diabetes on cardiac development by culturing hCOs in conditions of elevated glucose and insulin. These methodologies facilitate the rapid *in vitro* modeling of certain aspects of cardiac development. Nevertheless, the current applicability of these models is restricted to the initial stages of embryonic development. Consequently, there is a pressing need to advance the design of more precise cardiac organoids to establish highly accurate and reproducible culture models.

To date, the modeling of hCOs has proven effective in simulating pathological processes and cardiac development, serving as a complementary tool to preclinical models. However, hCOs have not yet fully supplanted characteristic parameters of animal models.

## 5 Toxicity testing and drug screening

### 5.1 Pharmacological testing based on various hCOs disease models

There is a high likelihood of adverse drug reactions in the heart and liver because they are particularly sensitive organs. Cardiotoxicity is responsible for 31% of adverse drug reactions (Wilke et al., 2007). There can be structural damage to cardiac tissue and impairment of cardiac function as a result of drug-induced cardiovascular toxicity, manifested as arrhythmias and altered cardiac contractility (Bowes et al., 2012). hiPSC-CM tissues have been shown to be excellent predictors of drug-induced arrhythmias and contractility by incorporating them (Peters et al., 2015). Beck et al. (2022) employed hCOs to evaluate the direct cardiotoxic effects of trametinib, observing a reduction in both the diameter and contractility of trametinib-treated hCOs in comparison to untreated controls, findings that align with existing *in vivo* data. Furthermore, the Cardiac Arrhythmia Suppression Test (CAST) has been developed to demonstrate the effectiveness of antiarrhythmic drugs in preventing ventricular arrhythmias associated with ischemic cardiomyopathy. However, some outcomes were attributed to an unforeseen increase in mortality due to arrhythmic events and shock. Kopljar et al. (2018) employed a calcium transient screening assay in cardiac cells derived from hiPSC-CMs to establish a hazard scoring system for assessing cardiac electrical liability. This methodology facilitated the integration of diverse pharmacological effects observed in hiPSC-CMs into a unified hazard label, thereby enabling the evaluation of antiarrhythmic drugs for potential drug toxicity. Sharma et al. (2017) employed hiPSC-CMs to assess cardiomyocyte viability, contractility, and changes in electrophysiology, calcium handling, and signaling pathways for screening the cardiotoxicity of tyrosine kinase inhibitors (TKIs). Based on these assessments, they developed a “cardiac safety index” to quantify the cardiotoxic potential of existing TKIs. They found that TKIs inhibited vascular endothelial growth factor receptor 2 (VEGFR2) and platelet-derived growth factor receptor (PDGFR) to cause cardiotoxicity in hiPSC-CMs, hiPSC-ECs, and hiPSC-CFs. Through phosphoprotein analysis, the researchers ascertained that the inhibition of VEGFR2/PDGFR by TKIs led to a compensatory enhancement in cardioprotective signaling pathways mediated by insulin and IGF in hiPSC-CMs. The upregulation of these cardioprotective pathways through the administration of exogenous insulin or IGF1 was found to enhance the viability of hiPSC-CMs during concurrent treatment with cardiotoxic TKIs targeting VEGFR2/PDGFR. Consequently, hiPSC-CMs serve as a viable model for detecting cardiovascular toxicity associated with anticancer TKIs, with the experimental findings demonstrating a correlation with clinical phenotypes.

### 5.2 Drug screening based on various hCOs disease models

Because of their accessibility, scalability, and genetic stability, organoids are extensively used in high-throughput drug screening. hCOs can replicate the microenvironment of the native heart, potentially making them superior models for preclinical drug screening. For instance, hiPSC-CMs have been employed to detect adverse reactions to targeted pharmaceuticals utilizing next-generation sequencing and proteomic analyses. A recent investigation utilized hydrogen peroxide to induce ischemic injury in hiPSC-CMs *in vitro*, leading to the identification of a novel small molecule compound that enhances the survival of hiPSC-CMs post-ischemic insult. This study underscores the potential for tissue-specific high-throughput screening of drug compounds derived from hiPSCs (Fiedler et al., 2020). Mills et al. (2019) employed the hCOs platform to evaluate 105 small molecules with regenerative potential. Their study revealed significant disparities between the hCO system and traditional 2D assays. Furthermore, hCO platforms are applicable for screening drugs that facilitate organoid growth, maturation, and function. The advancement of complex organoids to simulate organ-organ interactions has emerged as a critical objective in the screening of drug candidates. Skardal et al. (2020) developed a sophisticated multi-organ-on-a-chip platform, utilizing 3D organoid technology derived from primary stem cells. This platform not only sustains *in vitro* survival for a minimum of 28 days but also preserves long-term viability and functionality. A multi-organ-on-a-chip system was employed to screen drugs withdrawn by the Food and Drug Administration (FDA).

While hCOs serve as valuable models for drug testing, they remain significantly different from a natural heart. Enhancing the structural and functional resemblance of hCOs to the natural heart, alongside investigating the impact of the cardiac microenvironment and other organ systems on cardiac drug toxicity, could improve the reliability of drug screening and toxicity assessments (as shown in Figure 2).

## 6 Technical challenges and future perspectives

### 6.1 Complexity and standardization

Known as miniature and simplified *in vitro* models, organoids simulate the structural and functional attributes of organs and have garnered significant interest for possible applications in disease modeling, drug screening, and personalized medicine. However, they face many challenges, particularly when it comes to model complexity, data analysis, and standardization. Your choice of matrix material is crucial for your model construction (Jose et al., 2020; Xu et al., 2021; Kloxin et al., 2009). The advent of genome editing techniques has significantly advanced the development of homologous hPSCs, which are extensively utilized in two-dimensional disease models. In parallel, the integration of genome editing technologies enables precise modification of human cerebral organoids to correct mutations, thereby facilitating the creation of innovative and personalized therapeutic platforms for disease modeling. The CRISPR/Cas9 system represents a cutting-edge genome editing technology for the correction and mitigation of disease-associated mutations (Yang et al., 2018). Multidimensional interactions among diverse cell types are typically involved in the growth and development of organoids. The analysis and processing of multi-omics sequencing data presents substantial challenges to conventional analytical methodologies in organoid research (Zhou et al., 2023; Tang et al., 2023). In the broader field of data analysis, the absence of standardized protocols and real-time monitoring techniques exacerbates this issue, introducing considerable variability into the system. This variability is further intensified by manual data processing and subjective interpretations (Peng et al., 2018; Lukonin et al., 2021). As organoids increase in complexity, there is a pressing demand for the development of novel technologies to address the challenges of complexity and standardization inherent in organoid research. Recently, the advent of artificially intelligent organoids and artificial intelligence (AI) interfaces has the potential to expedite the development and clinical application of organoids by offering innovative insights and methodologies that could transform the field (Bai et al., 2024). Machine learning has emerged as a potent methodology for analyzing cellular electrochemical impedance spectroscopy measurements, demonstrating its capacity to distinguish between proliferative and differentiation behaviors. This capability holds significant potential for diverse applications in stem cell implantation therapies (Cunha et al., 2019). Kegeles et al. (2020) have developed a deep learning-based computational algorithm for the identification and prediction of retinal structures in stem cell-derived organoids using bright-field imaging. The differentiation of pluripotent stem cells into 3D organoids, representing the retina and other neural tissues, has emerged as a prominent *in vitro* strategy for modeling developmental processes. Meanwhile, Park et al. (2023) employed AI algorithms to predict the differentiation status of stem cells into kidney organoids, leading to the formation of more complex organoids. The integration of AI in organoid development holds significant potential for enhancing our understanding of the intricate biological systems inherent in various organoids. Furthermore, the emergence of multi-omics analyses, integrating genomic, transcriptomic, proteomic, and metabolomic data, has allowed researchers to model the development of human cerebral organoids with greater precision, while also shedding light on the intricate mechanisms underlying disease. According to Gu et al. (2022), several molecular events underlie the maturation and development of the developing heart based on an integrated multi-omics analysis. Their findings provide novel insights into the multilevel regulatory mechanisms governing developmental transitions.

### 6.2 Clinical translation and application prospects

In particular, AI technologies can be applied to optimize the design of matrix gels with desirable physicochemical properties (Suwardi et al., 2022; Verheyen et al., 2023). Moreover, the refinement of cell culture conditions through machine learning techniques holds the potential to diminish variability and minimize errors by automating the cell culture process. This automation facilitates the achievement of more consistent and reproducible results, while also supporting higher throughput (Kanda et al., 2022). Using extensive datasets of organoid-scale images, we can train machine learning algorithms to recognize and quantify diverse organoid features (Gritti et al., 2021). AI plays a pivotal role in the preclinical evaluation and application stages of organoid research. Through the utilization of predictive models and optimization algorithms, AI facilitates the assessment of mechanisms underlying organoid intervention development, the screening of potential pharmacological agents, and the construction of *in vitro* disease models. These applications are crucial for bridging the gap between fundamental research and clinical implementation (He et al., 2023; Kong et al., 2020; Monzel et al., 2020). The application of AI in multi-omics data analysis facilitates the integration of diverse functional information and the establishment of construction parameters. This approach enhances the efficiency and quality of organoid development, thereby expediting the progression from laboratory research to clinical applications.

## 7 Conclusion

The heart is an essential organ with critical functions, yet the complexities of cardiogenesis are not fully understood. Consequently, there is a pressing need to develop *in vitro* models of cardiac development and cardiovascular disease. hCOs have thus far demonstrated their value as complementary tools to *in vivo* studies. However, some challenges remain for the organoids. Firstly, the large-scale differentiation of organoid cells continues to present substantial challenges in terms of efficiency, quality, and orientation. Secondly, there is a pressing need to amalgamate advanced expertise in biology, materials science, and bioengineering to facilitate the construction of more precise and complex organ systems. Thirdly, the spontaneous formation of organoids relies on endogenous physiological mechanisms. The precise coordination of these signaling pathways during the developmental process is of paramount importance. It is essential to integrate these processes with validated methodologies, utilizing a range of established technologies, to advance hCOs technology from basic scientific applications to translational research with extensive and practical implications.

Facts:

• hCOs as an innovative model for CVD research, offering human-relevant insights.
•Applications in modeling myocardial infarction, heart failure, arrhythmias, and congenital heart diseases.
•Potential for drug screening and developing personalized therapeutic strategies.
•Integration of multi-omics and AI for improving model accuracy and clinical translation.
•Challenges in large-scale differentiation and model standardization.

Open questions:

•How to improve large-scale differentiation and reproducibility of hCOs?
•What are the key signaling pathways involved in hCO development?
•How to integrate bioengineering to enhance hCO complexity?
•Can hCOs be used for personalized medicine in CVD?
• What are the long-term implications of hCOs in drug testing and clinical research?
